# Ten years of Foodbook: Utilization of Foodbook survey data for research

**DOI:** 10.14745/ccdr.v51i67a06

**Published:** 2025-07-01

**Authors:** Heather Grieve, Jillian Macleod, Lauren E Grant

**Affiliations:** 1 Department of Population Medicine, Ontario Veterinary College, University of Guelph, ON

**Keywords:** Foodbook, public health, population health, foodborne disease, foodborne illness, enteric illness, epidemiologic methods, surveys, questionnaires

## Abstract

**Background:**

Enteric illnesses are a preventable cause of morbidity and healthcare utilization in Canada. To support public health and epidemiological activities, Foodbook was launched in 2014 by the Public Health Agency of Canada to collect representative information on food, water, and animal exposures, food safety knowledge, burden of gastrointestinal illnesses, and sociodemographic information. The aim of this overview was to identify how this valuable data source has been used in the past decade since its launch.

**Methods:**

Peer-reviewed and grey literature were identified by applying the search term “Foodbook” to two academic databases and two grey literature sources, respectively. Citations were screened against eligibility criteria. Study information, including study characteristics, module of Foodbook data used, and how Foodbook data was used was extracted and synthesized in tabular format.

**Results:**

A total of 27 articles were identified in the published literature that utilized Foodbook survey data in their analyses. The most common use was for outbreak investigations. In addition, Foodbook has been used to describe food, water, and animal exposures, determine food safety knowledge and practices of Canadians, estimate the burden of acute gastrointestinal illness, and evaluate data collection methods for foodborne illnesses.

**Conclusion:**

By summarizing its use, the authors aim to encourage broader use of this publicly available data source to inform health protection and promotion activities to reduce the burden of enteric illnesses in Canada.

## Introduction

Foodborne illnesses are a preventable cause of morbidity and healthcare utilization in Canada, with approximately 4 million annual episodes (1). While symptoms are often self-limiting, domestically acquired infections result in over 11,000 hospitalizations and 200 deaths each year ((2)). In Ontario alone, foodborne illnesses contribute to an estimated 137,000 primary care visits, 40,000 emergency department visits, and 6,200 hospitalizations annually, representing an important source of preventable healthcare utilization (3).

Foodbook is a population-based survey developed, conducted, and funded by the Public Health Agency of Canada (PHAC) to collect representative information on food, water, and animal exposures to support response to enteric illness outbreaks, inform health protection activities, and identify associations with sociodemographic factors and food safety behaviours ((4)). The first version of Foodbook (Foodbook 1.0) was conducted in all provinces and territories over a one-year period, from April 2014 to April 2015. The sampling frame consisted of cellphones (20%) and landlines (70% listed numbers plus 10% random digit dialing). Interviews were conducted in English, French, and Inuktitut and on demand translation was available for other languages. In addition to sociodemographic data and information on seven-day food consumption, information was also collected on other risk factors, including drinking and recreational water exposures, animal exposures, and food safety knowledge, as well as burden of acute gastrointestinal illnesses ((4)). Those who travelled outside the province or territory during the recall period were excluded. In the first cycle, 10,942 interviews were completed, with an overall response rate of 19.9%. A Foodbook 1.0 public use microdata file (PUMF) is available through the Government of Canada Open Government Portal ((5)).

Data collection for the second version (Foodbook 2.0) was completed from January 2023 to January 2024, online and by telephone, and the second report was released in July 2024 ((6)). This provides a timely opportunity to review how Foodbook data has been utilized to date. This article offers a brief synthesis describing the use of Foodbook data to advance our understanding of the epidemiology of enteric illnesses in the Canadian population, as available in the published literature.

## Methods

Peer-reviewed articles were identified by applying the search term “Foodbook” to two academic databases, PubMed and Web of Science, and two grey literature sources, namely Google Scholar and the PHAC website. No date or language limiters were applied. The initial search was conducted in July 2023 and updated in June 2024. Citations were screened according to the following inclusion criteria: 1) Foodbook was used as a data source and 2) the study was peer-reviewed. The initial list of included citations was then reviewed by a PHAC epidemiologist responsible for Foodbook to check for completeness. Study information, including study characteristics, module of Foodbook data used, and how Foodbook data was used was extracted and synthesized in tabular format.

## Results

In total, 27 articles were included that used Foodbook for enteric illness-related studies. These studies were broadly categorized into five purposes: outbreak investigations (n=11), exposure types (n=9), food safety knowledge and practices (n=3), methods development (n=3), and burden of acute gastrointestinal illnesses (n=1). An overview of these studies, including the modules of Foodbook utilized, a brief description of how the data was used, and the main findings is provided in **Table 1**.

**Table 1:** Summary of publicly available, peer-reviewed articles using Foodbook for enteric illness-related studies

Title (reference) (publication year)	Category	Study type	Foodbook module used	How Foodbook data was used	Main findings
An outbreak of *Salmonella* Typhimurium infections linked to ready-to-eat tofu in multiple health districts - Ontario, Canada, May–July 2021 ((7)) (2023)	Outbreak investigations	Descriptive	Food	Reference values for comparing reported food exposures against	Tofu was identified as the source of the outbreak.
Bi-national outbreak of *Salmonella* Newport infections linked to onions: the United States experience ((8)) (2022)	Outbreak investigations	Descriptive	Food	Reference values for comparing reported food exposures against	Red onions were identified as the source of the outbreak.
2015 outbreak of cyclosporiasis linked to the consumption of imported sugar snap peas in Ontario, Canada ((9)) (2017)	Outbreak investigations	Descriptive	Food	Reference values for comparing reported food exposures against	Fresh sugar snap peas imported from Guatemala were identified as the source of the outbreak.
*Escherichia coli* O121 outbreak associated with raw milk Gouda-like cheese in British Columbia, Canada, 2018 ((10)) (2021)	Outbreak investigations	Descriptive	Food	Reference values for comparing reported food exposures against	Raw milk Gouda-like cheese was found to be the source of the outbreak due to contaminated raw milk.
International outbreak of multiple *Salmonella* serotype infections linked to sprouted chia seed powder - USA and Canada, 2013–2014 (11) (2017)	Outbreak investigations	Descriptive	Food	Reference values for comparing reported food exposures against	Sprouted chia seed powder was the implicated source of the *Salmonella* outbreak.
Nuggets of wisdom: *Salmonella* Enteritidis outbreaks and the case for new rules on uncooked frozen processed chicken ((12)) (2017)	Outbreak investigations	Descriptive	Food	Reference values for comparing reported food exposures against	Uncooked, frozen, processed chicken products produced at a single establishment were implicated as the source of the outbreaks.
Fermenting a place in history: the first outbreak of *Escherichia coli* O157 associated with kimchi in Canada ((13)) (2023)	Outbreak investigations	Descriptive	Food, animal	Reference values for comparing reported food exposures against	Kimchi was implicated as the source of the outbreak, with Napa cabbage being the most likely contaminant.
Investigation of a *Salmonella* Montevideo outbreak related to the environmental contamination of a restaurant kitchen drainage system, Québec, Canada, 2020–2021 ((14)) (2023)	Outbreak investigations	Descriptive	Food	Reference values for comparing reported food exposures against	A restaurant was implicated as the likely source of the outbreak, though no source was conclusively identified.
Use of a case-control study and control bank to investigate an outbreak of locally acquired cyclosporiasis in Canada, 2016 ((15)) (2019)	Outbreak investigations	Case-control	Food	Reference values for comparing reported food exposures against, the Foodbook control bank was also used to recruit controls for a case-control study	Foodbook reference values highlighted several items consumed more frequently by cases, whereas the case-control study identified just two of these items as being consumed more frequently by cases. The source of the outbreak was not conclusively identified.
A multi-provincial *Salmonella* Typhimurium outbreak in Canada associated with exposure to pet hedgehogs, 2017–2020 ((16)) (2022)	Outbreak investigations	Descriptive	Animal	Reference values for comparing reported animal contact exposures against	Direct and indirect contact with hedgehogs was determined to be the source of the outbreak.
Outbreak of *Salmonella* Typhimurium associated with feeder rodents ((17)) (2018)	Outbreak investigations	Descriptive	Animal	Reference values for comparing reported animal contact exposures against	Feeder rodents were implicated as the main source of the outbreak.
Examining the diversity of ultra-processed food consumption and associated factors in Canadian adults ((18)) (2020)	Exposure types	Descriptive	Food	To describe ultra-processed food (UPF) consumption across Canada and explore associations between sociodemographic variables and UPF consumption	Most Canadians consume UPFs at least weekly. When controlling for potential confounders, younger age and higher BMI was associated with higher UPF consumption in both men and women.
Fast food consumption in adults living in Canada: alternative measurement methods, consumption choices, and correlates ((19)) (2023)	Exposure types	Descriptive	Food	To compare methods for estimating fast food consumption and to describe the fast-food consumption of Canadians, including any associations with sociodemographic variables	Asking detailed questions about fast food consumption results in increased recall compared to asking more broadly. Fast food consumption is common and some of the factors associated with increased consumption are gender-specific.
Country food consumption in Yukon, Northwest Territories and Nunavut, Foodbook study 2014–2015 ((20)) (2021)	Exposure types	Descriptive	Food	To describe consumption of country food amongst residents of Yukon, Northwest Territories and Nunavut	The consumption of specific country foods varied by territory, but inter-territory comparisons are difficult due to differences in landscape, climate, populations, and cultural factors. Generally, country food consumption increased with age and lower income was associated with higher consumption.
Consumption of high-risk foods in the Canadian population, Foodbook study, 2014 to 2015 (21) (2021)	Exposure types	Descriptive	Food	To describe consumption of high-risk foods amongst the Canadian population	Consumption of high-risk foods is common in Canadians. The number of high-risk foods consumed was similar amongst males and females, but the types of high-risk food consumed varied by gender. Knowledge of risk did not appear to impact on consumption.
Drinking and recreational water exposures among Canadians: Foodbook study 2014–2015 ((22)) (2018)	Exposure types	Descriptive	Water	To describe drinking and recreational water exposures amongst the Canadian population	Drinking water sources differ between provinces with private well use being greater in the Maritime provinces than in the rest of the country. Recreational water exposures are highest amongst children aged 0–9 years.
Measuring animal exposure in Canada: Foodbook study, 2014–2015 ((23)) (2018)	Exposure types	Descriptive	Animal	To describe animal exposure, including direct and indirect contact, amongst the Canadian population	Cats and dogs were the most commonly reported animal exposures. Children aged 0–9 years reported relatively high exposure to higher risk animals such as rodents and reptiles.
Risk profile of hepatitis E virus from pigs or pork in Canada ((24)) (2017)	Exposure types	Descriptive	Food	To estimate pork and pork liver consumption amongst Canadians to inform development of a risk profile for hepatitis E virus from pigs or pork	The proportion of the population at high risk for acquiring hepatitis E from pigs or pork in Canada is considered to be relatively small.
A comparative exposure assessment of *Campylobacter* in Ontario, Canada ((25)) (2017)	Exposure types	Descriptive	Food, water, animal	To inform frequency estimates for modelling exposure to *Campylobacter* via food, water, and animal contact routes	The results suggest that some transmission routes such as raw milk and companion animal contact are underestimated in the existing literature.
A comparative exposure assessment of foodborne, animal contact and waterborne transmission routes of *Salmonella* in Canada ((26)) (2020)	Exposure types	Descriptive	Food, water, animal	To inform frequency estimates for modelling exposure to *Salmonella* via food, water, and animal contact routes	Chicken meat was the highest exposure route.
Canadian consumer food safety practices and knowledge: Foodbook study ((27)) (2017)	Food safety knowledge and practices	Descriptive	Consumer food safety	To describe the food safety practices and knowledge of Canadians	The majority of Canadians take appropriate cleaning and separating precautions to prevent foodborne illness, however, use of food thermometers is low. Differences in practices and knowledge were found between some sociodemographic groups, including gender and age.
Identifying predictors of safe food handling practices among Canadian households with children under eighteen years ((28)) (2023)	Food safety knowledge and practices	Descriptive	Food safety	To identify determinants of safe food handling amongst Canadian households with children younger than 18 years old.	Key differences in food safety practices were found between different sociodemographic groups, including education levels, and living in an urban area.
Predictors of safe food handling among Canadian seniors living at home ((29)) (2020)	Food safety knowledge and practices	Descriptive	Food safety	To identify determinants of safe food handling practices amongst Canadian seniors living at home	Most seniors followed instructions and food labels and appropriately refrigerated food. Women and younger seniors were more likely to have better food handling practices.
Online population control surveys: A new method for investigating foodborne outbreaks ((30)) (2020)	Methods development	Descriptive	Food	Online survey data collected during an outbreak investigation was compared to Foodbook data to evaluate the use of online surveys as a method for collecting data during outbreaks	Online surveys allow for rapid collection of control data which can be used in outbreak investigations, whilst also providing flexibility about what data is collected.
The use of an online survey for collecting food exposure information, Foodbook sub-study, February to April 2015 (31) (2021)	Methods development	Descriptive	Food	To compare food exposure data collected by online surveys to that collected via telephone survey	Reported food consumption was higher for those completing the online survey compared to the telephone survey.
Comparison of 3-day and 7-day recall periods for food consumption reference values in foodborne disease outbreak investigations ((32)) (2019)	Methods development	Descriptive	Food	A sub-sample of Foodbook respondents were asked about food exposures during the past 3 days (compared to 7 days for the main study). The food exposure frequencies for the two groups were compared	The majority of food consumption frequencies were similar for both groups, however, when applied during an outbreak investigation only the three-day recall period reference values supported the conclusion that chicken was the source of the outbreak.
The incidence of acute gastrointestinal illness in Canada, Foodbook survey 2014–2015 ((33)) (2017)	Burden of gastrointestinal illness	Descriptive	Acute gastrointestinal illness	To estimate the incidence of acute gastrointestinal illness in the last 28 days and describe healthcare seeking behaviours	It was estimated that there are 0.57 self-reported acute gastrointestinal illness episodes per person-year, and less than 10% of cases seek medical care.

Abbreviations: BMI, body mass index; UPF, ultra-processed food

## Discussion

The most common use of Foodbook is to support outbreak investigations. There are several examples of outbreak investigations successfully using Foodbook food exposure data as reference values to which the reported exposures of cases are compared, including a *Salmonella* Typhimurium outbreak linked to pre-prepared tofu ((7)), *Salmonella* Newport infections linked to onions ((8)), cyclosporiasis linked to sugar snap peas ((9)), an *Escherichia coli* outbreak linked to raw milk Gouda-style cheese ((10)), *Salmonella* infections of multiple serovar types linked to sprouted chia seed powder ((11)), and *Salmonella* Enteritidis infections linked to frozen, uncooked, processed chicken ((12)).

In an *Escherichia coli* outbreak investigation, comparisons were made to the Foodbook reference values and significant differences were observed; however, the source of the outbreak was determined to be kimchi, which is not included in the Foodbook data ((13)). In another study, reference values for food exposures were used during a *Salmonella* Montevideo outbreak ((14)). While the source was later attributed to contaminated plumbing at a restaurant, it was suggested that the plumbing could have been contaminated by chicken, and indeed, cases were significantly more likely to have consumed chicken at a restaurant compared to the reference values provided by Foodbook.

An investigation into a cyclosporiasis outbreak utilized Foodbook for both reference values and for recruiting controls for a case-control study ((15)). This was made possible by creating a control bank of Foodbook participants who consented to being contacted to assist with future investigations. The authors highlight that having access to this control bank allowed for a case-control study to be conducted in a timely and cost-effective manner.

Two outbreaks of *Salmonella* Typhimurium utilized Foodbook as part of their investigations, where hedgehogs ((16)) and feeder rodents ((17)) were eventually identified as the respective causes, demonstrating that Foodbook can be used to investigate outbreaks with animal sources.

## Characterizing different exposures

### Food exposures

Collection of food exposure information has elucidated the eating patterns of Canadians. For example, ultra-processed foods (UPF) are commonly consumed, with these items being consumed at least weekly by 99.0% of respondents ((18)). Younger age and higher body mass index (BMI) were associated with UPF consumption in both males and females. Fast-food was consumed at least weekly by 48.0% of respondents, with highest fast-food consumption among men and those of younger age ((19)). Sex-specific multivariable logistic regression models highlighted differences between males and females. For example, women in central Canada (compared to the territories), and men with an income of $30,000 to $80,000 (versus a higher income) had higher fast-food consumption ((19)).

Foodbook respondents from Yukon (YT), Northwest Territories (NT) and Nunavut (NU), were also asked questions regarding consumption of country or traditional food items, referring to “food that is trapped, fished, hunted, harvested, or grown for subsistence or medicinal purposes, outside of the commercial food chain” ((34)). Consumption of country food during the previous seven days varied by territory, ranging from 60.7% of respondents in NT to 77.5% in NU ((20)). Overall, this food-specific data can support nutrition and food security research, in addition to outbreak investigations ((20)).

Consumption of foods considered high-risk for enteric illness, such as unpasteurized milk, cheese, or juice, is common among Canadians, with approximately 94% of respondents consuming at least one high-risk food per week, and more than half consuming three or more foods ((21)). Importantly, knowledge of high-risk foods did not influence consumption, suggesting that improving food safety practices may be of greater benefit in reducing the risk of illness associated with these foods.

### Water exposures

One study analyzed Canadians’ exposure to drinking and recreational water ((22)). While most Canadians use municipal drinking water (68.5%), 10.8% of respondents indicated that a private well was their primary source of drinking water; however, this figure was as high as 40.2% to 55.6% in the Maritime provinces. Private well use was also highest among rural residents. Further, 18.8% of respondents used bottled water as their primary source, and residents of Saskatchewan (SK) were more likely to consume bottled water ((22)). Regarding recreational water, children aged 0–9 years were most exposed. Unsurprisingly, recreational water exposure was highest across all age groups during the summer months, during which up to 30% of respondents reported recreational water exposure. Together, this data informs private well water testing recommendations and messaging, as well as enhanced public health messaging during the summer months, with specific messaging aimed at parents and caregivers given the higher level of recreational water use among children ((22)).

### Animal exposures

A descriptive analysis of animal exposure data showed that most respondents (63.4%) had contact with an animal, animal food or waste, or an animal habitat within the previous seven days ((23)). Animal contact was highest among children, an at-risk group for more serious enteric illness. Overall, dogs and cats were shown to be the most common animal exposure. Given that companion animals are often an overlooked source of zoonotic infections, this analysis provides the basis for continued awareness campaigns to reduce the risk of illness and improved surveillance for identifying outbreaks.

### Comparative exposure assessments

Comparative exposure assessments have been conducted utilizing Foodbook to characterize different exposure routes to hepatitis E from pigs or pork ((24)), *Campylobacter* ((25)) and *Salmonella* ((26)). In the first study, the number of Canadians consuming pork liver supported the hypothesis that a relatively low number of Canadians are at high risk of contracting hepatitis E via this route ((24)). Foodbook was used more extensively in the *Campylobacter* and *Salmonella* studies. In both studies, chicken meat was the highest source of foodborne exposures, and the authors discuss how interventions could reduce risk posed by these pathogens ((26)).

### Food safety knowledge and practices

Foodbook has been used to explore the food safety knowledge and practices of Canadians ((27)), including those at higher risk for severe enteric illness, namely, children ((28)) and seniors ((29)). While most Canadians reported cleaning their hands after handling meat and taking appropriate measures to avoid cross contamination, the use of food thermometers and awareness of specific high-risk foods was low, highlighting the need for improved messaging and education ((27)).

Most seniors followed instructions on food labels and refrigerated leftovers within two hours ((29)). Following instructions on food labels was significantly associated with food safety storage practices. Interestingly, there was no association between knowledge that seniors are at an increased risk of foodborne illness and safe food storage practices, highlighting that food safety knowledge is not an accurate proxy for food safety practices among Canadian seniors ((29)).

Among households with children, higher earners, and those living in urban areas were less likely to wash their hands with soap after handling raw meat ((28)). The practice of separating food in the refrigerator to avoid cross contamination was more likely to be practiced by female caregivers and single-child households. Education level was inversely associated with food safety practices, as those with a bachelor’s degree were less likely to refrigerate food within two hours and use a food thermometer. These findings demonstrate key demographic subgroups that may benefit from targeted public health messaging relating to food safety practices.

### Methods development in foodborne illness research

Using the Foodbook telephone survey data as a comparator group, the utility of online surveys for collecting food exposure information was investigated by two studies ((30,31)), which found that online surveys present an efficient, accurate, and lower cost approach for data collection, which may be particularly useful during outbreak investigations when speed of response is important. Differences in exposure information collected online and by telephone may partially be explained by differing recall periods (14-day for the online survey versus 7-day for Foodbook) and changing food preferences ((30)).

The impact of recall periods on reported food exposures and their subsequent utility in identifying outbreak sources was examined by one study, which found no overall difference in reported consumption of most foods when comparing three- and seven-day recall periods ((32)). However, when data from a *Salmonella* Infantis outbreak was compared to the reference values for both groups, only the three-day recall period group showed a significant result for the identified source of the outbreak, suggesting that a three-day recall period is preferred when investigating illnesses with a short incubation period or where the source is a commonly consumed item ((32)).

A study discussed earlier, which utilized Foodbook data to explore fast food consumption, also demonstrated that question type can influence the reported consumption of fast foods ((19)). In the Foodbook survey, there are three questions relating to fast food consumption; in one question, no examples of fast food were given, whereas in two more detailed questions, examples of specific fast-food types were provided. Respondents who answered the broader question reported less consumption of fast foods compared to those answering either of the two more detailed questions ((19)).

### Estimating the burden of acute gastrointestinal illness

Analysis of Foodbook data provided the first estimate of acute gastrointestinal illness burden in Canada, with an estimated 19.5 million episodes each year ((33)). Differences in burden were demonstrated (albeit not significantly) between provinces and territories, highlighting the need to collect nationally representative data. This information, coupled with demonstrated seasonal patterns, can be used in the design of locally tailored public health interventions, and in healthcare services planning ((33)).

## Limitations

### Foodbook 1.0

The Foodbook 1.0 report lists several limitations, including potential non-response bias due to the exclusion of individuals without a telephone and those unable to communicate in one of the survey languages. This may have disproportionately impacted residents of Northern Canada, where there is a larger proportion of individuals living in remote communities without reliable access to a telephone, affecting representativeness of the data and resultant findings. Other types of bias outlined in the report include recall bias and low response rate ((4)).

Several of the included articles identified additional limitations of Foodbook, including recall bias ((21−23,33)) and non-response bias ((18-22,27,28,33)). Several studies discussed the potential for social desirability bias, including in relation to consumption of UPFs ((18)), fast-food ((19)), high risk foods ((21)) and food handling practices ((28,29)). The lack of demographic variables, including ethnicity, pregnancy status, and health status (i.e., whether an individual is considered high risk for enteric illness) also limits exploration of food consumption in particular groups ((20,21,27)). The small sample size for some sub-populations, such as parents and caregivers, was also highlighted as a limitation ((28)), and the availability of data regarding education level ((22)). Further, the sample size of respondents selected to participate in the online survey sub-study ((31)) and three-day recall period sub-study ((32)) were highlighted as limitations.

The utility of Foodbook exposure data is also limited by the specific foods included in the study ((10,13,14,18,21)). This includes lack of information relating to serving size or frequency of intake, which is of particular importance when attempting to quantify exposure to particular food groups ((18)). For one study, the seven-day recall period selected by Foodbook presented a limitation as this did not match with case interviews, where a 14-day period was chosen to align with the incubation period for cyclosporiasis ((15)). In terms of animal exposure, Foodbook did not capture data regarding where the animal exposure occurred, meaning that it is not possible to draw conclusions about high-risk animal environments ((23)). Lastly, data for the food safety practices module were collected from November to April, and this was identified as a limitation given the potential for food safety practices to vary over time ((27)).

### Foodbook 2.0

Foodbook 2.0 includes several key updates. First, a dual sampling frame was utilized with 75% of the sample derived from a list of mailing addresses and the remainder from listed telephone numbers ((6)). To increase the number of children participating, after the fifth month of data collection in households with children, a child was selected to participate 100% of the time. Ethnicity data was also collected as part of Foodbook 2.0.

Additional exposure data included information on special diets, food shopping habits, and information relating to types of water and animal exposures. We also note that additional food items identified as potential sources of enteric illness are included in Foodbook 2.0, expanding the utility of Foodbook as a resource for use in outbreak investigations ((6)).

## Conclusion

Foodbook is a valuable data resource for food, water, and animal exposures, food safety behaviours, and socio-demographic factors that increase risk of enteric illnesses in Canada. This resource has supported multiple public health activities, most predominantly, outbreak investigations. In this commentary, we have focused on the use of Foodbook as described in the published literature. However, it is recognized that other uses of Foodbook may not have resulted in publications. By synthesizing published studies, the aim is to increase the visibility of Foodbook as an openly accessible resource for conducting epidemiological studies. In particular, the release of Foodbook 2.0 provides an opportunity to assess how patterns in food, water, and animal-related exposures have changed over time and to update associations with socio-demographic and behavioural factors. Moreover, there is an opportunity to utilize Foodbook to inform food safety and health promotion activities. Lastly, linkage to other data sources, application of a health equity lens to assess differential risk across key socio-demographic groups, and novel use of methodological tools, such as artificial intelligence, will provide further insights to reduce enteric illness burden in Canada.
